# Supplementary material for: The interactome of intact mitochondria by cross-linking mass spectrometry provides evidence for coexisting respiratory supercomplexes
Source: Mol Cell Proteomics. 2017 Dec 8;17(2):216–32. doi: 10.1074/mcp.RA117.000470 (PMC5795388; doi:10.1074/mcp.RA117.000470)
Supplement: Supplemental Data [file supp_RA117.000470_133922_0_supp_23978_fzffwf.zip › spectra_annotation/mito_DR_spectra_annotation/281-1-3-1-5-1.pdf]

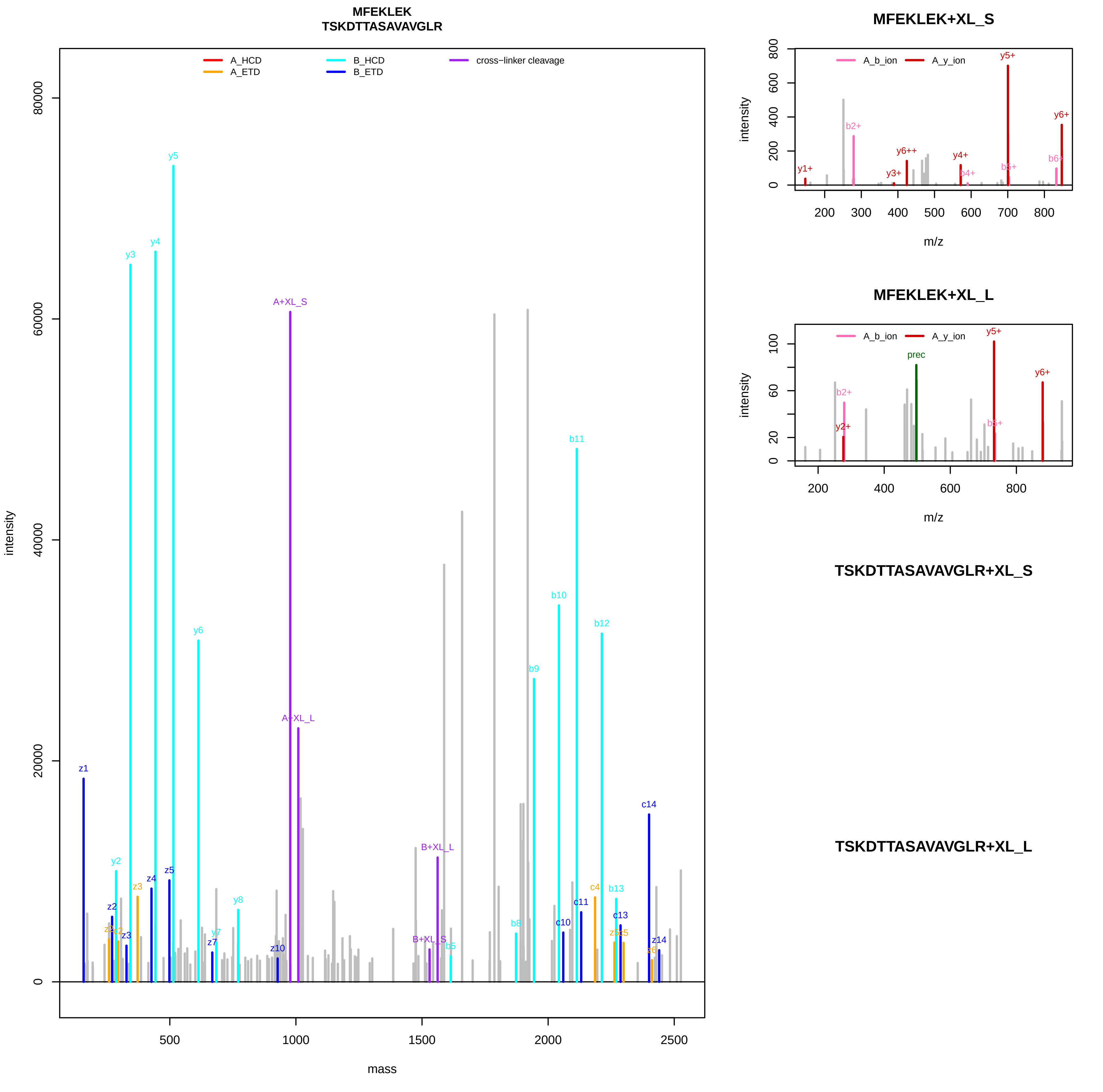
MFEKLEK
TSKDTTASAVAVGLR
A_HCD
A_ETD
B_HCD
B_ETD
cross−linker cleavage
intensity
mass
0
20000
40000
60000
80000
500
1000
1500
2000
2500
z1
y2
z2
z3
y3
z3
y4
z4
y5
z5
y6
z7
y7
y8
z10
A+XL_S
A+XL_L
B+XL_S
B+XL_L
b5
b8
b9
b10
c10
b11
c11
b12
c4
b13
c13
c14
z14
MFEKLEK+XL_S
A_b_ion
A_y_ion
y1+
b2+
y3+
y6++
y4+
b4+
y5+
b5+
b6+
y6+
intensity
m/z
0
200
400
600
800
200
300
400
500
600
700
800
MFEKLEK+XL_L
A_b_ion
A_y_ion
prec
b2+
y2+
b5+
y5+
y6+
intensity
m/z
0
20
60
100
200
400
600
800
TSKDTTASAVAVGLR+XL_S
TSKDTTASAVAVGLR+XL_L
